# Syndrome of Inappropriate Secretion of Antidiuretic Hormone (SIADH) With Severe Hyponatremia As the Initial and Sole Presentation of COVID-19 Infection: A Case Report and Pathophysiologic Insights

**DOI:** 10.7759/cureus.34161

**Published:** 2023-01-24

**Authors:** Parinaz Ayat, Sultana Alam, Violeta Capric, James Chen, Samy I McFarlane

**Affiliations:** 1 Department of Medicine, Downstate-Health Science University, Brooklyn, USA; 2 Department of Internal Medicine: Endocrinology, Diabetes and Metabolism, Downstate-Health Science University, Brooklyn, USA

**Keywords:** hyponatremia, atypical presentation, sars-cov-2 infection, covid-19, siadh

## Abstract

Hyponatremia is one of the common electrolyte imbalances among hospitalized patients with the syndrome of inappropriate secretion of antidiuretic hormone (SIADH) being a common etiology for hyponatremia. There are multiple pathophysiologic considerations in the differential diagnosis of the etiologic factor for SIADH, including infections such as pneumonia and meningitis, as well as coronavirus disease 2019 (COVID-19) infection. However, SIADH, as the sole initial presentation of the infection of COVID-19, is rarely reported. In this report, we present a case of SIADH as the initial and only presentation of a COVID-19 infection, highlighting the clinical course and treatment strategy while providing the putative pathophysiologic insights into this unusual and potentially serious complication of COVID-19 infection.

## Introduction

Since December 2019, the whole world has been facing one of the most serious health crises in recorded history, caused by a virus from the beta-coronavirus family. Named coronavirus disease 2019 (COVID-19), it is responsible for the severe acute respiratory syndrome coronavirus 2 (SARS-CoV-2) pandemic. The first confirmed case of COVID-19 was reported in the United States on January 31, 2020, with acute hypoxemic respiratory failure being the most common hospitalization diagnosis with COVID-19.

This virus mostly presents with upper or lower respiratory tract illness, with fever, congested nose, cough, dyspnea, and myalgia being the most common clinical presentations, with up to 5% developing severe illness leading to multiorgan damage [1,2].

Hyponatremia is one of the most common electrolyte abnormalities in hospitalized patients and is associated with a higher rate of mortality [3]. One of the most common causes of hyponatremia is the syndrome of inappropriate secretion of antidiuretic hormone (SIADH) with diagnostic criteria that include sodium (Na) less than 135 mmol/l, increased urine osmolality more than 100 mOsm/kg, with salt and normal fluid intake, euvolemia without signs of hypo or hypervolemia, serum osmolarity less than 275 mOsm/kg, and urine Na more than 40 mEq/L [4].

Etiologically, SIADH can be attributed to multiple factors, including tumors (paraneoplastic syndrome), infections such as pneumonia and meningitis, and disorders of the nervous system such as stroke and subarachnoid hemorrhage [5]. SIADH can also be caused by several pharmacological agents, including anticonvulsants, antipsychotics, antidepressants, and cytotoxic drugs [6].

Several infections can be associated with SIADH; however, SIADH caused by COVID-19 infection has rarely been reported as the sole presentation of this disease [4]. In this report, we describe a case of SIADH as the initial and sole presentation of COVID-19 infection in a 62-year-old man with an acute mental status change.

## Case presentation

A 62-year-old man with a past medical history of hypertension and type 2 diabetes mellitus was brought into the emergency department with acute mental status change and a complaint of chest pain that started one night prior to admission. The patient’s vital signs were within normal limits, and he appeared euvolemic; the physical exam was remarkable for altered mental status, where he was disoriented but without meningeal signs or any other significant finding, but shortly after the emergency department presentation patient experienced a tonic-clonic seizure with initial laboratory values remarkable with a glucose level of 264 mg/dl and a serum sodium level of 116 mEq/L and COVID-19 positive. Electrocardiogram showed sinus tachycardia and no ischemic changes, Chest X-ray with no acute pathology and the head computer tomography scan (CTS) reported vascular calcification and no acute pathological finding. The patient is started on hypertonic saline (3%) at 60 ml/hour and admitted to the medical intensive care unit (MICU) for further management. In the MICU, the saline infusion was changed to 2%, and his sodium level improved to 124 mEq/L (Table 1). Subsequently, the saline infusion was discontinued, his mental status improved, and he could tolerate an oral diet and was started on a fluid-restricted diet.

**Table 1:** Laboratory values, including initial presentation, and hospital course, including serum and urine electrolytes, as well as serum and urine osmolality, demonstrated improvement of the electrolytes profile with the administration of 3% saline to a safe, above 120 meq/dl in the first 24 hours *Sample was taken at 1 am, so the result is within the normal limit. ** Sample was taken at 8 am. BUN: blood urea nitrogen; TSH: thyroid-stimulating hormone

Lab Element (Normal Value)	Admission day	Day 2	Day 4	Day 5	Discharge Day
Serum Na mEq/L (135-145mEq/L)	116mEq/L	125mEq/L	129mEq/L	133mEq/L	134mEq/L
Serum K mmol/L (3.5-5.5 mmol/L)	3.6mmol/L	3.9mmol/L	4.1mmol/L	4.0mmol/L	4.3mmol/L
Serum Cl mEq/L (96- 106 mEq/L)	79mEq/L	92mEq/L	94mEq/L	97mEq/L	97mEq/L
Serum BUN mg/dL (6-24 mg/dL)	11mg/dL	13mg/dL	16mg/dL	16mg/dL	15mg/dL
Serum Cr mg/dL (0.74-1.35mg/dL)	0.93mg/dL	0.88mg/dL	1.08mg/dL	1.25mg/dL	1.13mg/dL
Serum Osm mOsm/kg (275-295 mOsm/kg)	251mOsm/kg	267mOsm/kg			
Urine Osm mOsm/kg (300-1000 mOsm/kg)	441mOsm/kg	192mOsm/kg		213mOsm/kg	369mOsm/kg
Urine Na mEq/L (20 mEq/L in a random urine sample)	93mEq/L			<20mEq/L	21mEq/L
Urine K mEq/L (20 mEq/L in a random urine sample)	31.5mEq/L			14mEq/L	31.3mEq/L
Urine Cl mEq/L (25-40 mEq/L)	37mEq/L			<20mEq/L	<20mEq/L
Urine Cr mg/dL (39-259 mg/dL)				107mg/dL	88.44mg/dL
TSH uIU/ml (0.270-4.20 mIU/L)	0.849 uIU/ml			1.320 uIU/ml	
Cortisol level ug/dL (6.20-19.40ug/dL)	3.39ug/dL^* ^				18.30ug/dL^*^

Laboratory findings (Table 1) showed serum osmolality was 251, which improved to 267(mOsm/Kg) with the administration of 3% saline, and his urine osmolality also improved from 441 to 192 (mOsm/Kg). Brain magnetic resonance imaging (MRI) reported no acute findings. The patient also had normal thyroid and adrenal profiles with TSH of 0.85 uIU/ml and fasting serum cortisol of 18.3ug/dl. A diagnosis of SIADH was made, with COVID-19 infection being the only identifiable etiology for hyponatremia. The patient’s hypoosmolar hyponatremia improved by 3%, followed by 2% saline administration, and his mental status improved to baseline. He was deemed medically stable for discharge home on Day 6 of the admission with an outpatient follow-up with primary care physician.

## Discussion

COVID-19 has a wide range of symptoms, with pneumonia and acute hypoxemic respiratory failure being the most common diagnosis in hospitalized patients. A few case reports documented SIADH in the setting of pneumonia; However, the exact mechanism of hyponatremia development is not fully understood; several mechanisms have been postulated including pain and psychological, emotional, or physical stresses associated with COVID-19 infection could possibly stimulate hypothalamohypophyseal axis-induced ADH release [5]. Other mechanisms include impaired osmoregulation induced by inflammatory factors such as Interlukin-6 (IL-6). Further potential mechanisms, such as hypoxemia and nausea, have also been proposed [7]. COVID-19 infection itself has been recognized as a hyper-inflammatory state and could induce excessive cytokine release, which is one of the causes of SIADH, and, consequently, hyponatremia [7]. There are two mechanisms postulated for cytokine-induced hyponatremia; one is direct stimulation of the hypothalamohypophyseal and, subsequently, the nonosmotic release of antidiuretic hormone (ADH) [5] or ventilation-perfusion mismatch induced by alveolar cell injury secondary to cytokines release, which can cause SIADH as a result of inadequate left atrium filling secondary to hypoxic pulmonary vasoconstriction [5,7].

Another hypothesis for COVID-19-related SIADH is that positive pressure ventilation (PPV) could cause ADH secretion due to nonosmotic stimulation [8]. In adult patients with COVID-19 pneumonia, 71% required mechanical ventilation, making PPV a potentially causative factor that should be considered in this population with hyponatremia [8].

## Conclusions

Although the relationship between COVID-19 and SIADH still needs further investigation, in the presence of SIADH with no other diagnosed pathology, as highlighted in this case report, COVID-19 itself should be considered as the diagnostic etiology for SIADH since it could be the initial and even the sole clinical presentation of COVID-19 infection. With this in mind, COVID-19 treatment protocols, as well as acute management of hyponatremia, could help manage those patients with COVID-19-related SIADH, a potentially serious disease entity with increased mortality.
